# Defining reference values for the gut microbiota in a Southern European population

**DOI:** 10.3389/fcimb.2026.1766733

**Published:** 2026-05-12

**Authors:** Chiara Pollicardo, Franca Gotta, Paolo Bottino, Daria Vay, Valentina Pizzo, Sara Scaglione, Giulia Pontarollo, Marialuisa Novi, Lara Boatti, Flavio Mignone, Giovanni Melioli, Fabio Rapallo, Andrea Rocchetti

**Affiliations:** 1 Department of integrated Surgical and Diagnostic Science (DISC), University of Genova, Genova, Italy; 2 Alliance Medical Diagnostic Ltd, Genova, Italy; 3 Microbiology and Virology Laboratory, University Hospital “SS. Antonio e Biagio e C. Arrigo”, Alessandria, Italy; 4 Department of Translational Medicine, Eastern Piedmont University, Novara, Italy; 5 Gastroenterology Unit, University Hospital “SS. Antonio e Biagio e C. Arrigo”, Alessandria, Italy; 6 Arrow Diagnostics Ltd, Genova, Italy; 7 SmartSEQ Ltd, Alessandria, Italy; 8 Phenomix Ltd, Genova, Italy; 9 Department of Economics, University of Genova, Genova, Italy

**Keywords:** 16S, fecal microbiota transplantation, gut microbiota, healthy population, references values, sequencing, Southern Europe

## Abstract

**Introduction:**

The clinical implementation of gut microbiota analysis requires the definition of reliable reference values derived from standardized and certified methodologies applied to a population representative of the intended clinical setting.

**Methods:**

In this study, 250 fecal samples were analyzed using a CE-certified 16S rRNA bacterial profiling assay for taxonomic characterization across multiple bacterial levels. Sequencing reads were quality-filtered and aligned against the RDP database (release 11, update 5); only sequences with ≥80% alignment coverage and ≥97% similarity were retained.

**Results:**

The resulting taxonomic distributions were first compared with data previously obtained from a similar population, revealing only minor differences. As an additional validation, comparative analyses were performed with data from a clinical study on fecal microbiota transplantation (FMT). Donor profiles were highly consistent with those obtained in the present study, whereas pre-transplant samples showed marked deviations from the reference ranges; post-transplant profiles progressively converged toward them.

**Discussion:**

Although the limited sample size precludes accurate assessment of rare taxa (<0.1% relative abundance), the use of a single Next-Generation Sequencing (NGS) platform and the focus on a Southern European population characterized by the Mediterranean diet allowed the establishment of the first set of gut microbiota reference values derived from a certified *in-vitro* diagnostic workflow. These data represent an essential step toward the integration of gut microbiota testing into clinical laboratory practice, enabling standardized interpretation of microbiota imbalance and supporting targeted medical interventions aimed at restoring microbial equilibrium.

## 1 Introduction

The gut microbiota represents a complex community of microorganisms inhabiting the human digestive tract, playing several roles in digestion, immune regulation and overall health (Khalil et al., 2024). Alterations in its composition have been associated with several gastrointestinal disorders, including inflammatory bowel disease (Crohn’s disease and ulcerative colitis) (Kirkik and Kalkanli Tas, 2024), irritable bowel syndrome (Jauregui-Amezaga and Smet, 2024), *Clostridioides difficile* infection (Normington et al., 2024), colorectal cancer (Smyth et al., 2024), celiac disease (Catassi et al., 2024), small intestinal bacterial overgrowth (SIBO) (Ghoshal et al., 2023), and diverticulitis (Cameron et al., 2023). Beyond the gastrointestinal tract, dysbiosis has also been linked to various systemic and extraintestinal conditions, such as metabolic disorders (obesity and type 2 diabetes) (Enache et al., 2024), cardiovascular disease and atherosclerosis (Ronen et al., 2024), autoimmune disorders (rheumatoid arthritis and multiple sclerosis) (Qusty et al., 2024), allergic diseases (asthma and atopic dermatitis) (Rios-Carlos et al., 2024), neurological and psychiatric disorders, including Parkinson’s and Alzheimer’s diseases (Kustrimovic et al., 2024; Özaydin Aksun et al., 2024), autism spectrum disorder, depression and anxiety (Bogielski et al., 2024), as well as hepatic conditions such as non-alcoholic fatty liver disease (NAFLD) and cirrhosis (Garcia-Mateo et al., 2024). Given these associations, it is evident that the gut microbiota exerts a pivotal influence on human health and disease conditions. Its composition and function are shaped by multiple external factors, particularly diet. Indeed, diets rich in fat, sugar, and animal-based or highly processed foods (typical of Western dietary patterns) can favor the proliferation of harmful bacteria, leading to dysbiosis and inflammation (Brichacek et al., 2024). On the contrary, high-fiber and plant-based diets, as well as the consumption of probiotics and fermented foods, promote microbial diversity and short-chain fatty acid production, thereby enhancing intestinal homeostasis and overall health (Bavaro et al., 2024; Dimba et al., 2024). Environmental and lifestyle factors such as antibiotic use, urbanization, pollution, stress, hygiene, and sanitation also further contribute to inter-individual variability in gut microbial profiles (Filardo et al., 2022).

In recent years, microbiota analysis has become increasingly relevant in clinical practice. However, the field remains marked by substantial methodological heterogeneity. Differences in DNA extraction protocols, primer design, library preparation, choice of 16S rRNA variable regions, sequencing technologies, analytical depth and bioinformatics pipelines can overall influence the microbial profiles obtained (Fernández-Pato et al., 2024). Many assays currently used in clinical laboratories are classified as *laboratory-developed tests* (LDTs), defined as *in vitro* diagnostic tests designed, manufactured, and used within a single laboratory (U.S. Food and Drug Administration (FDA), 2025). Others are commercially produced but limited to *Research Use Only* (RUO). Only a few are CE-certified *in vitro diagnostic* (IVD) tests, suitable for diagnostic use in clinical settings. When IVD tests are employed, adherence to medical laboratory reporting standards is mandatory (European Commission, 2023), including reference ranges, as also strongly suggested (Porcari et al., 2024).

Reference ranges also referred to as reference values (R.V.), must be specific to the analytical method used and derived from a population representative of the intended reference group. For gut microbiota analysis, dietary habits should be as homogeneous as possible, since applying reference values from populations with markedly different diets may introduce bias. In clinical contexts, gut microbiota reference values are essential not only for the identification of dysbiosis but also for guide of nutritional interventions, probiotic or prebiotic administration, therapeutic strategies and preventive measures. Given the vast complexity of the gut microbiota (encompassing dozens of phyla, hundreds of genera and thousands of species), the technical challenges and costs of next-generation sequencing (NGS), as well as the natural variability in taxa proportions (Sisti et al., 2022), reference values should ideally be derived from a single CE-certified method and based on robust statistical approaches.

When possible, these values can be established by analyzing data from healthy controls. However, even among apparently healthy individuals, marked inter-individual variability is often observed (Sisti et al., 2022; Fontana et al., 2019). Unlike biochemical parameters, which typically follow narrow distributions, microbiota components exhibit broad variability (Sisti et al., 2022). Therefore, alternative approaches, such as those employed in pediatric studies where healthy cohorts are difficult to recruit (Cangemi et al., 2012), can be applied. These methods rely on the assumption that samples obtained from the general community during routine testing adequately represent the reference population. In community medicine, extreme alterations of the microbiota are rare - contrary to hospital or specialized settings - allowing for the calculation of mean, median, standard deviation, and percentile intervals for most taxa, except for those present at very low frequencies.

In the present work, we analyzed stool samples processed in our laboratory to determine the distribution of bacterial taxa at different taxonomic levels, from phylum to species. We also report the corresponding frequency distributions and reference intervals, providing a detailed overview of gut microbiota composition in a Southern European population.

## 2 Materials and methods

### 2.1 Sample size

To estimate the minimum microbial prevalence that can be detected in a set of *n* biological samples with a given probability *P*, the following probabilistic model was applied. Assuming that the presence of a microbe in the population follows a Bernoulli process, the probability of *not* detecting the microbe in any of the *n* independent samples is given by:


$$P_{none}={\left(1-p\right)}^{n}$$

where p represents the true prevalence of the microorganism in the population.

Consequently, the probability of detecting the microorganism at least once in n samples is:


$${\text{P}}_{\text{at}\;\text{least}\;\text{one}}\;=\;1-{\left(1-p\right)}^{\text{n}}$$

Given a fixed sample size (*n* = 250) and a desired detection probability (*P* ≥ 0.95), the following inequality must hold:


$$1-{\left(1-p\right)}^{\text{n}}\geq \;0.95$$

Rearranging yields:


$${\left(1-p\right)}^{n}\leq 0.05$$

$$\log(1-p)\leq \frac{\log(0.05)}{n}$$

$$1-p\leq 10^{\frac{\log(0.05)}{n}}\Rightarrow p\geq 1-10^{\frac{\text{log}(0.05)}{n}}$$

Substituting *n* = 250, the minimum detectable prevalence (*p*) is approximately 1.2% for a detection probability of ≥95%. From a microbiological perspective, detecting a microbial population present in as little as 1.2% of fecal samples may still be clinically relevant, particularly in the context of infectious diseases. Under the analytical conditions used in this study, with an average sequencing depth of approximately 100000 reads per sample, the theoretical minimum detectable frequency (corresponding to a single read) is 0.001%. In this way, however, signals supported by very few reads (1–5) are often considered statistically unreliable due to stochastic variation and potential sequencing artifacts. Therefore, adopting a more conservative detection threshold of ≥10 reads, the realistic minimum detectable frequency ranges between 0.01% and 0.05%, regardless of the total number of samples analyzed.

### 2.2 Fecal samples

A total of 250 fecal samples were retrieved from laboratory records of individuals who underwent routine gut microbiota analysis performed outside the hospital setting. None of these samples originated from subjects with known severe gastrointestinal or colorectal disorders, such as active-phase inflammatory bowel disease, gastrointestinal bleeding, or colorectal cancer. Among the analyzed specimens, 68% were from women and 32% from men. The age distribution was the following: 12% were under < 30 years (12%), 30–50 years (33%), 50–70 years (38%), >70 years (17%).

Each sample was collected using DNA/RNA Shield tubes (Zymo Research; Euroclone, Milan, Italy), following the manufacturer’s instructions, and stored at −20 °C until processing.

Being a purely laboratory-based study, only limited clinical information, mainly age and sex, was available. Nevertheless, samples from patients with known severe gastrointestinal symptoms and/or laboratory test results suggesting specific diseases were excluded. These included alterations in lipid profiles suggestive of obesity, elevated fasting glucose levels indicative of diabetes, or increased liver enzyme concentrations suggesting liver disease.

In addition, samples showing macroscopic alterations of the fecal microbiota profile - such as a marked increase in Negativicutes - were also excluded.

Finally, the vast majority of subjects undergoing fecal microbiota analysis completed a questionnaire reporting their dietary habits. These data were compared with the recommendations of the Mediterranean Diet Foundation (MDF, Bach-Faig et al., 2011).

For quality control purposes, during the study period a fecal sample was aliquoted, stored frozen, and periodically extracted and analyzed together with routine samples.

An internal quality control (QC), consisting of the repeated analysis of a single stool sample aliquoted into multiple tubes and stored frozen, was performed once per month throughout the two-year study period.

As an external control, gut microbiota data from fecal microbiota transplantation (FMT) donors and recipients were obtained from the Microbiology and Virology Laboratory of the University Hospital. Alessandria University Hospital. Samples were collected under an approved clinical trial (CE approval AOO-ISS-08/06/2022-0021984; Class: CNT 01.00) and processed using the analytical workflow. Microbiota profiles were analyzed from 18 donors, 19 pre-transplant recipients (T0), and 16, 11, and 9 recipients at 7, 30, and 60 days post-transplantation, respectively. Donor samples were expected to closely match the established reference values, whereas pre-transplant recipient samples were expected to show marked deviations from them.

### 2.3 DNA extraction and sequencing

Microbial DNA was extracted from 0.2 mL of Universal transport medium containing the fecal sample using a SeePrep32 automated extractor with STARMag 96 ProPrep reagents (Seegene, Arrow Diagnostics, Italy). Following extraction, DNA concentration was quantified using a Qubit Fluorometer (Thermo Fisher Scientific, Italy) in order to stardardize the subsequently steps.

Next-generation sequencing (NGS) of the bacterial 16S rRNA gene was performed targeting the V3–V4–V6 hypervariable regions, using the Microbiota Solution B Kit (Arrow Diagnostics Srl, Genoa, Italy), in accordance with the manufacturer’s instructions. To our knowledge, this kit is among the few commercially available solutions CE-certified for *in vitro* diagnostic (IVD) use. Briefly, the target regions were amplified by target PCR. After validation by agarose gel electrophoresis using an E-Gel Power Snap System (Thermo Fisher Scientific), amplicons were subjected to index PCR to assign a unique index to each sample for downstream sequencing. Following normalization and pooling, libraries were sequenced on an Illumina^®^ MiSeq™ or Iseq 100 platforms (Illumina Inc., San Diego, CA, USA).

### 2.4 Bioinformatics and data analysis

Raw data were uploaded in a dedicated bioinformatics system for taxonomic classification and analysis using MicrobAT^®^ software (*Microbiota Analysis Tool*, SmartSeq Srl, Italy), which is included in the CE certification for *in vitro* diagnostic (IVD) use. In the initial processing phase, sequencing reads were filtered using a proprietary algorithm to remove short and low-quality sequences. The remaining high-quality reads were then aligned against the Ribosomal Database Project (RDP) reference database, release 11, update 5, for taxonomic assignment. Only sequences meeting the predefined quality criteria—alignment length ≥80% of the reference sequence and similarity ≥97%—were assigned to the corresponding species-level taxon. The resulting dataset provided both the absolute read counts and the relative abundances of bacterial taxa across multiple taxonomic ranks, including phyla, classes, orders, families, genera, and species.

### 2.5 Statistical analysis

Taxonomic abundances were expressed as relative percentages prior to percentile computation. The abundance data were organized in a matrix format, with samples represented as rows and taxa as columns. For each taxon, the 5^th^, 15^th^, 25^th^, 50^th^, 75^th^, 85^th^ and 95^th^ percentiles of its distribution across all samples were calculated using the quantile() function in R (version 4.3.2). Percentile estimates were obtained using the default type-7 method for quantile estimation, excluding missing values (na.rm = TRUE). Differences in taxonomic distributions between female and male subjects were assessed using the Wilcoxon rank-sum test. Data manipulation and visualization were performed with the R packages tidyr, dplyr, and ggplot2. The same analytical routines were applied to compare taxa distributions at the phylum, family, and genus levels with those reported in a matched reference population. A similar approach was also used to describe and compare the frequencies of phyla between donors and recipients of fecal microbiota transplantation, relative to the reference values established in the present study.

## 3 Results

In order to evaluate the dietary habits of the study population, the results of the questionnaire were compared with the dietary recommendations of the MDF. The Mediterranean diet emphasizes the consumption of fruits, vegetables, whole grains, legumes, nuts, seeds, and olive oil, together with a moderate intake of fish, poultry, and red wine (Abenavoli et al., 2024) The results of this comparison are reported in **Table 1**. A substantial overlap was observed between the reported dietary habits and the recommendations of the MDF. This finding suggests that the study population, although randomly collected and subject to only minor exclusion criteria, was relatively homogeneous with regard to dietary habits.

**Table 1:** Weekly frequency of consumption of major food groups in the study population compared with expected frequencies of a Mediterranean dietary pattern.

Food group	Median (days/week)	Mediterranean reference (days/week)	Adherence to Mediterranean pattern
Fruit	7	7	within
Nuts	3	3–7	within
Vegetables	6	7	below
Pasta	4	3–7	within
Bread	3.5	5–7	below
Red meat	2	0–1	above
White meat	2.5	1–3	within
Fish	2	2–4	within
Legumes	2	2–3	within
Cheese	5	1–3	above
Eggs	2	2–3	within
Milk	5.5	3–7	within
Sweets	4	0–1	above
Wine	1.5	0–7	within

Consumption is expressed as median days/week. Mediterranean reference ranges were derived from commonly accepted Mediterranean diet recommendations reported in the literature (PMID: 22166184).

Across samples, at phylum level (**Table 2**) the intestinal microbiota was dominated by Firmicutes and Bacteroidetes, which together accounted for the majority of the relative abundance. Firmicutes showed a median relative abundance of 36.4% (IQR: 31.6–44.8%), while Bacteroidetes were slightly lower, with a median of 32.3% (IQR: 27.4–41.5%). The third most abundant phylum was Proteobacteria (median 2.5%), followed by Actinobacteria (0.7%) and Verrucomicrobia (0.02%), which displayed higher variability among samples, with some reaching up to ~9%. Minor phyla such as Fusobacteria, Lentisphaerae, and Tenericutes were detected sporadically and generally contributed less than 1% to the total community, although Tenericutes occasionally reached >5% in some outlier samples. Phyla represented at a frequency lower than 20% (which include Synergistetes, Euryarchaeota, Planctomycetes, Cyanobacteria Chloroplast, Spirochaetes, Acidobacteria, SR1, Armatimonadetes, Aquificae, Chloroflexi and Deinococcus-Thermuswere) were not shown. The quality control procedures implemented during the study period indicated good reproducibility of the entire analytical workflow, from fecal DNA extraction to bioinformatic analysis. At the phylum level, highly prevalent taxa such as Firmicutes and Bacteroidetes showed limited variability (CV: 7.7% and 7.1%, respectively). Less prevalent taxa showed greater variability.

**Table 2:** Distribution of reference values for major Phyla of the intestinal microbiota: prevalence, mean abundance and percentile distribution.

Taxa	Positive (%)	Mean	2.5%	5%	10%	25%	50%	75%	90%	95%	97.5%
Firmicutes	100.00	46.261	21.157	24.986	28.232	31.576	36.377	44.840	56.331	60.842	63.304
Bacteroidetes	100.00	39.994	1.614	8.801	22.960	27.432	32.326	41.523	49.841	52.997	55.247
Proteobacteria	100.00	6.209	0.339	0.791	1.730	1.989	2.501	4.536	7.565	9.733	11.509
Actinobacteria	100.00	3.516	0.095	0.147	0.316	0.421	0.691	1.968	4.814	6.385	8.644
Verrucomicrobia	85.66	1.548	0.002	0.004	0.006	0.010	0.021	0.159	1.374	2.891	5.217
Fusobacteria	68.88	0.496	0.001	0.001	0.003	0.005	0.012	0.044	0.130	0.229	0.393
Lentisphaerae	61.19	0.308	0.001	0.001	0.002	0.004	0.017	0.105	0.303	0.560	0.846
Crenarchaeota	57.69	0.006	0.001	0.001	0.001	0.002	0.002	0.003	0.005	0.008	0.009
Candidatus Saccharibacteria	43.36	0.039	0.001	0.002	0.002	0.003	0.003	0.006	0.012	0.022	0.030
Tenericutes	31.82	1.853	0.001	0.001	0.002	0.003	0.004	0.012	0.173	1.891	5.738
Synergistetes	28.32	0.110	0.001	0.002	0.002	0.003	0.005	0.021	0.058	0.108	0.141
Euryarchaeota	18.88	0.017	0.001	0.001	0.001	0.001	0.002	0.005	0.010	0.016	0.032
Elusimicrobia	1.05	0.253	0.006	0.007	0.009	0.010	0.014	0.023	0.377	0.518	0.589
Armatimonadetes	0.70	0.002	0.002	0.002	0.002	0.002	0.002	0.002	0.002	0.002	0.002

At the class level (**Table 3**), Clostridia (median 28.8%) and Bacteroidia (median 32.5%) were the dominant taxa, reflecting the prevalence of Firmicutes and Bacteroidetes, respectively. Other frequent microbial classes included Gammaproteobacteria (median 0.13%), Actinobacteria (median 0.73%), and Negativicutes (median 1.02%). Verrucomicrobiae exhibited a wide distribution range (0.08%–17.46%), suggesting that this taxon, although typically minor, may become relatively abundant in certain individuals, likely due to the expansion of Akkermansia. Mollicutes (median 0.004%) and Erysipelotrichia (median 0.25%) were present at low but non-negligible levels. Classes represented at a frequency lower than 20% (which include Epsilonproteobacteria, Chloroplast, Planctomycetia, Sphingobacteriia, Spirochaetia, Cyanobacteria, SR1 genera incertae sedis, Thermoplasmata, Acidobacteria Gp3, Aquificae, Anaerolineae) were not shown. At the class level, *Bacteroidia* and *Clostridia* were also reproducible (CV: 7.6% and 6.3%, respectively). The CV slightly increased for less frequent bacteria as well as for taxonomic levels reported below (orders, families, genera, and species). Nevertheless, well-represented taxa showed CV values <10%, whereas less frequent taxa showed CV values <20%. Considering the complexity of the analytical workflow, these results indicate acceptable reproducibility of the method during the two-year study period. Data about quality control are available in the “additional result” section of the online version of this manuscript.

**Table 3:** Distribution of reference values for major classes of the intestinal microbiota: prevalence, mean abundance and percentile distribution.

Taxa	Positive (%)	Mean	2.5%	5%	10%	25%	50%	75%	90%	95%	97.5%
Clostridia	100.000	37.976	1.236	8.940	20.449	24.468	28.804	38.941	48.934	52.828	54.963
Bacilli	100.000	4.635	0.030	0.039	0.089	0.113	0.155	0.438	1.373	3.206	6.556
Actinobacteria	100.000	3.675	0.101	0.155	0.334	0.435	0.735	2.083	4.927	6.750	9.047
Negativicutes	100.000	2.923	0.030	0.170	0.529	0.653	1.025	1.923	3.827	5.258	6.043
Gammaproteobacteria	100.000	2.309	0.017	0.026	0.047	0.075	0.131	0.406	2.076	4.143	6.679
Bacteroidia	99.301	40.604	0.813	15.20	22.721	26.842	32.463	41.646	51.184	53.879	56.633
Erysipelotrichia	98.951	1.558	0.025	0.061	0.109	0.161	0.255	0.629	1.879	3.416	4.581
Betaproteobacteria	98.601	2.479	0.011	0.029	0.149	0.390	0.917	1.848	3.396	4.658	5.198
Deltaproteobacteria	94.755	0.701	0.003	0.006	0.032	0.115	0.218	0.540	0.931	1.197	1.361
Verrucomicrobiae	83.916	1.636	0.002	0.004	0.005	0.008	0.018	0.167	1.305	3.073	5.456
Alphaproteobacteria	81.818	0.797	0.002	0.002	0.004	0.005	0.011	0.040	0.325	0.513	0.728
Fusobacteriia	68.881	0.531	0.001	0.001	0.003	0.005	0.012	0.047	0.138	0.243	0.421
Lentisphaeria	60.490	0.326	0.001	0.002	0.003	0.005	0.019	0.123	0.323	0.606	0.914
Thermoprotei	57.692	0.006	0.001	0.001	0.001	0.002	0.002	0.004	0.006	0.008	0.010
Flavobacteriia	51.399	0.098	0.001	0.002	0.003	0.003	0.005	0.013	0.034	0.060	0.099
Epsilonproteobacteria	45.804	0.270	0.001	0.001	0.002	0.003	0.004	0.007	0.015	0.028	0.055
Saccharibacteria i. sedis	43.357	0.042	0.001	0.002	0.002	0.003	0.003	0.006	0.013	0.023	0.032
Mollicutes	31.818	1.986	0.001	0.001	0.002	0.003	0.004	0.013	0.183	2.097	6.100
Opitutae	31.469	0.124	0.001	0.002	0.002	0.003	0.004	0.017	0.123	0.264	0.357
Synergistia	28.322	0.121	0.001	0.002	0.002	0.003	0.006	0.022	0.059	0.110	0.154
Chloroplast	17.133	0.029	0.001	0.001	0.002	0.002	0.003	0.008	0.021	0.034	0.071
Spirochaetia	15.035	0.071	0.002	0.002	0.002	0.002	0.003	0.004	0.009	0.017	0.038

In **Table 4** were reported the values at order level: the most represented orders were Clostridiales (median 29.2%) and Bacteroidales (median 30.7%), followed by Enterobacteriales (median 0.03%) and Selenomonadales (median 0.85%). The Verrucomicrobiales order, consistent with the class-level findings, showed substantial inter-individual variability (0.01–15.6%). Desulfovibrionales and Burkholderiales were also present at low to moderate levels (medians 0.51% and 2.25%, respectively), indicating potential functional roles in sulfur and nitrogen metabolism within the gut environment. Orders that represented at a frequency lower than 20% (which include Synergistales, Campylobacterales, Planctomycetales, Neisseriales, Chloroplast, Aeromonadales, Sphingobacteriales, Desulfurococcales, Caulobacterales, Bdellovibrionales, Rhodobacterales, Subdivision5 genera incertae sedis, Rhodospirillales, Mycoplasmatales, Spirochaetales, SR1 genera incertae sedis, Thermoplasmatales, Anaerolineales, Candidatus Carsonella, Cardiobacteriales, Chromatiales, Family I, Gp3, Halanaerobiales, Myxococcales, Thiotrichales, Alteromonadales, Desulfobacterales, Xanthomonadales, Vibrionales, Thermales, Spartobacteria genera incertae sedis, Rhodocyclales) were not shown.

**Table 4:** Distribution of reference values for major order of the intestinal microbiota: prevalence, mean abundance and percentile distribution.

Taxa	Positive (%)	Mean	2.50%	5%	10%	25%	50%	75%	90%	95%	97.5%
Lactobacillales	83 .217	5 .223	0 .033	0 .070	0 .097	0 .161	0 .444	1 .890	8 .950	22 .216	84 .499
Selenomonadales	83 .217	2 .800	0 .026	0 .135	0 .516	0 .853	1 .713	3 .442	5 .891	8 .053	11 .191
Coriobacteriales	83 .217	1 .794	0 .010	0 .029	0 .104	0 .288	0 .800	2 .736	4 .816	5 .996	7 .145
Clostridiales	82 .867	38 .785	1 .134	7 .064	18 .609	29 .202	40 .987	49 .632	56 .112	59 .289	64 .933
Bacteroidales	82 .517	39 .080	0 .921	8 .772	21 .479	30 .709	39 .670	50 .635	56 .813	62 .107	64 .265
Erysipelotrichales	82 .168	1 .595	0 .023	0 .067	0 .108	0 .257	0 .688	2 .085	4 .916	6 .069	7 .512
Burkholderiales	80 .769	2 .102	0 .009	0 .025	0 .131	0 .816	1 .624	2 .993	4 .688	5 .527	6 .974
Actinomycetales	80 .769	0 .162	0 .003	0 .004	0 .006	0 .016	0 .046	0 .092	0 .153	0 .402	0 .644
Enterobacteriales	79 .720	1 .744	0 .007	0 .009	0 .012	0 .033	0 .234	1 .221	6 .013	8 .974	16 .181
Bifidobacteriales	77 .622	2 .055	0 .008	0 .014	0 .026	0 .177	0 .901	2 .482	5 .454	8 .139	12 .895
Desulfovibrionales	77 .622	0 .581	0 .005	0 .011	0 .050	0 .208	0 .490	0 .825	1 .215	1 .768	2 .135
Pasteurellales	68 .182	0 .390	0 .002	0 .003	0 .005	0 .015	0 .070	0 .262	0 .827	1 .959	2 .743
Verrucomicrobiales	67 .483	1 .407	0 .001	0 .004	0 .005	0 .030	0 .222	1 .474	5 .645	9 .087	15 .601
Fusobacteriales	54 .895	0 .420	0 .001	0 .001	0 .004	0 .014	0 .052	0 .151	0 .442	1 .343	6 .456
Victivallales	49 .301	0 .178	0 .001	0 .002	0 .003	0 .013	0 .100	0 .279	0 .812	1 .095	1 .562
Bacillales	48 .951	0 .032	0 .001	0 .001	0 .002	0 .004	0 .007	0 .014	0 .065	0 .142	0 .937
Flavobacteriales	40 .210	0 .044	0 .001	0 .002	0 .003	0 .005	0 .014	0 .047	0 .112	0 .142	0 .368
Sphingomonadales	33 .217	0 .090	0 .001	0 .001	0 .002	0 .003	0 .006	0 .015	0 .052	0 .259	0 .402
Campylobacterales	30 .070	0 .144	0 .001	0 .001	0 .002	0 .003	0 .006	0 .011	0 .076	0 .736	3 .628
Neisseriales	30 .070	0 .082	0 .001	0 .002	0 .002	0 .005	0 .018	0 .040	0 .059	0 .087	1 .323
Pseudomonadales	25 .170	0 .001	0 .001	0 .001	0 .002	0 .003	0 .005	0 .015	0 .089	0 .247	0 .276

At the family level (**Table 5**), the most abundant taxa were *Ruminococcaceae* (median 12.2%), *Lachnospiraceae* (11.4%) and *Bacteroidaceae* (12.8%), all associated with fiber degradation and short-chain fatty acid (SCFA) production. *Prevotellaceae* (median 0.13%, 75th percentile: 3.35%) showed marked variability, suggesting the presence of diet-associated clustering. *Erysipelotrichaceae* (median 0.28%), *Veillonellaceae* (0.11%) and *Enterobacteriaceae* (0.04%) were less abundant but widely distributed across samples. The mucin-degrading *Verrucomicrobiaceae* showed a low median abundance (0.019%) but occasionally reached over 14%, further supporting the variability of *Akkermansia* presence among individuals. Families with frequencies lower than 30% (including, among others, *Bacillales i.s. XI, Saccharibacteria g.i.sOxalobacteraceae, Propionibacteriaceae, Bradyrhizobiaceae, Leuconostocaceae, Methanobacteriaceae, Anaeroplasmataceae, Staphylococcaceae, Peptococcaceae 1, Saccharibacteria genera incertae sedis, Pseudomonadaceae, Flavobacteriaceae, Puniceicoccaceae, Synergistaceae, Campylobacteraceae, Corynebacteriaceae, Planctomycetaceae, Comamonadaceae, Chloroplast, Gracilibacteraceae, Neisseriaceae, Aerococcaceae, Leptotrichiaceae, Phyllobacteriaceae, Syntrophomonadaceae, Incertae Sedis XI, Caulobacteraceae, Clostridiales Incertae Sedis XI, Sphingomonadaceae, Bacillales Incertae Sedis XI, Enterococcaceae, Peptococcaceae1, Succinivibrionaceae, Moraxellaceae, Spirochaetaceae*) were not shown.

**Table 5:** Distribution of reference values for major families of the intestinal microbiota: prevalence, mean abundance and percentile distribution.

Taxa	Positive (%)	Mean	2.5%	5%	10%	25%	50%	75%	90%	95%	97.5%
Lachnospiraceae	100.000	19.907	0.478	3.896	6.967	9.369	11.433	18.575	28.016	31.567	34.467
Ruminococcaceae	99.650	17.245	0.114	0.468	4.544	8.862	12.248	17.723	22.957	25.605	27.387
Coriobacteriaceae	99.650	1.765	0.018	0.032	0.100	0.164	0.270	0.749	2.461	3.905	4.800
Erysipelotrichaceae	98.951	1.729	0.027	0.066	0.117	0.187	0.285	0.686	2.117	3.806	5.145
Bacteroidaceae	98.601	21.742	0.064	0.754	4.980	8.971	12.854	20.792	29.639	34.922	38.624
Porphyromonadaceae	98.601	5.967	0.057	0.150	1.226	1.975	2.764	4.816	8.167	10.388	12.057
Enterobacteriaceae	97.552	1.973	0.006	0.009	0.014	0.019	0.044	0.272	1.354	3.390	5.723
Veillonellaceae	97.552	1.685	0.009	0.016	0.025	0.040	0.115	0.749	1.914	2.569	3.582
Prevotellaceae	97.203	9.871	0.019	0.029	0.053	0.078	0.139	3.359	15.497	24.143	30.617
Rikenellaceae	97.203	2.954	0.019	0.060	0.209	0.332	0.752	1.853	3.944	5.344	6.679
Streptococcaceae	96.503	1.172	0.010	0.013	0.027	0.045	0.079	0.212	0.767	1.960	2.826
Desulfovibrionaceae	94.056	0.630	0.003	0.007	0.033	0.120	0.205	0.507	0.801	1.030	1.324
Peptostreptococcaceae	93.706	0.903	0.003	0.007	0.013	0.021	0.036	0.167	1.090	1.809	2.436
Bifidobacteriaceae	93.357	2.217	0.006	0.014	0.028	0.046	0.158	0.909	2.635	3.716	5.635
Sutterellaceae	91.958	2.349	0.003	0.006	0.098	0.261	0.726	1.733	3.132	4.337	5.145
Acidaminococcaceae	88.811	1.492	0.004	0.005	0.009	0.013	0.032	0.684	2.263	3.428	4.231
Verrucomicrobiaceae	83.916	1.855	0.002	0.004	0.006	0.009	0.019	0.196	1.470	3.523	6.112
Pasteurellaceae	83.916	0.460	0.002	0.003	0.005	0.008	0.015	0.067	0.244	0.443	0.817
Clostridiaceae	79.371	0.423	0.002	0.003	0.005	0.007	0.013	0.086	0.401	0.721	0.870
Actinomycetaceae	77.972	0.052	0.002	0.002	0.004	0.005	0.007	0.016	0.039	0.056	0.077
Carnobacteriaceae	73.427	0.062	0.001	0.002	0.002	0.003	0.005	0.011	0.025	0.036	0.053
Fusobacteriaceae	65.734	0.392	0.001	0.002	0.004	0.006	0.011	0.042	0.120	0.224	0.319

At the genus level (**Table 6**), the most dominant taxa included *Bacteroides* (median 17.15%), *Faecalibacterium* (median 5.6%), and *Prevotella* (median 0.84%, up to 46.1% in some samples), highlighting a bimodal distribution typical of *Bacteroides*- vs *Prevotella*-dominated gut communities. Other relevant genera were *Blautia* (median 0.21%), *Ruminococcus* (median 0.26%), *Alistipes* (median 0.97%) and *Parabacteroides* (median 1.72%). *Akkermansia* (median 0.25%, up to 19.4%) stood out for its inter-individual variability, confirming its heterogeneous distribution in the population. *Bifidobacterium* and *Streptococcus* were less dominant (medians 0.16% and 0.09%, respectively). Only taxa present in at least 50% of stool samples were shown.

**Table 6:** Distribution of reference values at genus level of the intestinal microbiota: prevalence, mean abundance and percentile distribution.

Taxa	Positive (%)	Mean	2.5%	5%	10%	25%	50%	75%	90%	95%	97.5%
*Faecalibacterium*	98.951	10.280	0.050	0.089	0.505	1.901	5.625	9.923	13.859	16.831	19.249
*Bacteroides*	98.601	27.744	0.090	0.954	6.914	11.426	17.152	28.501	37.345	42.610	45.864
*Blautia*	96.853	3.860	0.047	0.064	0.096	0.142	0.217	0.786	6.987	9.783	11.136
*Parabacteroides*	96.503	4.053	0.009	0.128	0.960	1.186	1.728	3.068	5.321	6.689	8.742
*Alistipes*	96.154	3.617	0.023	0.097	0.280	0.456	0.974	2.330	4.844	6.847	8.006
*Butyricicoccus*	96.154	0.367	0.009	0.012	0.030	0.048	0.075	0.201	0.487	0.757	0.918
*Lachnospiracea incertae sedis*	95.455	3.925	0.151	0.349	0.516	1.025	1.904	3.493	5.184	6.042	6.875
*Oscillibacter*	95.105	1.695	0.013	0.052	0.201	0.335	0.567	1.308	2.418	3.203	3.894
*Streptococcus*	95.105	1.303	0.010	0.015	0.028	0.049	0.088	0.243	0.947	2.085	2.992
*Flavonifractor*	95.105	0.338	0.019	0.029	0.057	0.067	0.117	0.218	0.404	0.586	0.700
*Gemmiger*	94.406	1.408	0.004	0.007	0.033	0.112	0.375	0.906	1.799	2.821	3.585
*Clostridium XlVa*	94.406	0.948	0.054	0.082	0.122	0.194	0.295	0.581	1.332	1.864	2.188
*Dorea*	94.056	0.878	0.011	0.036	0.062	0.097	0.134	0.407	1.325	1.879	2.256
*Roseburia*	92.308	1.349	0.005	0.015	0.057	0.119	0.345	0.947	1.890	2.756	3.056
*Clostridium IV*	92.308	0.671	0.006	0.010	0.029	0.045	0.074	0.239	0.734	0.964	1.355
*Bilophila*	91.958	0.494	0.003	0.008	0.035	0.084	0.172	0.375	0.692	0.863	0.964
*Barnesiella*	90.210	2.016	0.004	0.009	0.020	0.047	0.234	1.267	2.504	4.195	4.930
*Collinsella*	89.510	1.745	0.004	0.008	0.036	0.093	0.176	0.632	2.368	4.087	5.165
*Odoribacter*	89.510	0.711	0.003	0.008	0.072	0.137	0.280	0.522	0.850	1.154	1.423
*Bifidobacterium*	88.462	2.069	0.009	0.012	0.026	0.061	0.160	1.020	2.480	3.791	5.733
*Coprococcus*	88.462	0.653	0.003	0.007	0.027	0.065	0.134	0.457	0.878	1.209	1.395
*Ruminococcus*	87.762	2.143	0.006	0.014	0.052	0.106	0.264	0.904	3.218	5.071	6.095

Finally, at the species level (**Table 7**), the core microbiota was represented by *Faecalibacterium prausnitzii* (median 3.1%), *Bacteroides vulgatus* (median 0.42%), *Bacteroides dorei* (median 0.21%), and *Bacteroides uniformis* (median 0.17%), all known butyrate or propionate producers contributing to intestinal homeostasis. Other abundant species included *Parabacteroides distasonis*, *Alistipes putredinis* and *Bacteroides ovatus*. *Akkermansia muciniphila* was not directly listed but inferred from the *Akkermansia* genus distribution, suggesting its presence in a subset of samples. Pathobionts such as *Escherichia/Shigella* (up to 13.7%) and *Streptococcus salivarius* (up to 5.8%) appeared sporadically at high abundance, indicating occasional dysbiotic patterns. Only species present in at least 50% of laboratory samples are available in this table. The complete list of not shown genera and species is available in the “additional result” section of the online version of this manuscript.

**Table 7:** Distribution of reference values at species level of the intestinal microbiota: prevalence, mean abundance and percentile distribution.

Taxa	Positive (%)	Mean	2.5%	5%	10%	25%	50%	75%	90%	95%	97.5%
*Faecalibacterium prausnitzii*	95.804	6.373	0.011	0.020	0.323	1.215	3.067	6.068	9.098	10.935	11.990
*Bacteroides* sp. *WA1*	94.056	3.390	0.007	0.039	0.228	0.438	0.902	2.283	4.977	6.214	7.530
*Bacteroides dorei (T)*	93.007	5.236	0.005	0.006	0.013	0.024	0.208	1.725	7.714	11.603	16.149
*Bacteroides vulgatus*	92.657	3.686	0.009	0.013	0.035	0.057	0.420	2.118	5.124	7.652	9.348
*Bacteroides uniformis*	92.657	0.828	0.013	0.027	0.063	0.086	0.177	0.450	0.911	1.339	1.703
*Bacteroides thetaiotaomicron*	92.657	0.583	0.006	0.015	0.036	0.057	0.111	0.283	0.728	0.985	1.396
*Flavonifractor plautii*	90.909	0.122	0.009	0.013	0.021	0.026	0.036	0.085	0.146	0.207	0.242
*Bacteroides vulgatus ATCC 8482*	90.559	1.972	0.004	0.007	0.013	0.031	0.131	0.656	2.375	4.228	6.010
*Oscillospiraceae bacterium AIP 1035.11*	90.210	0.421	0.015	0.026	0.050	0.067	0.119	0.263	0.579	0.722	0.901
*Blautia faecis (T)*	89.860	0.689	0.004	0.008	0.013	0.022	0.044	0.151	0.901	1.955	2.352
*butyrate-producing bacterium M21/2*	89.510	2.125	0.002	0.007	0.060	0.119	0.639	1.694	3.151	3.984	4.637
*Parabacteroides distasonis*	89.510	1.679	0.003	0.075	0.216	0.321	0.552	1.154	2.380	3.005	3.621
*Faecalibacterium prausnitzii (T)*	89.510	0.430	0.002	0.005	0.012	0.021	0.055	0.182	0.598	0.838	1.010
*Streptococcus salivarius subsp. null*	88.462	0.993	0.007	0.012	0.018	0.028	0.050	0.192	0.798	1.371	2.822
*Bacteroides ovatus*	87.063	1.094	0.005	0.016	0.030	0.066	0.112	0.457	1.105	1.875	3.000
*Clostridium clostridioforme*	87.063	0.705	0.006	0.007	0.017	0.038	0.068	0.240	0.862	1.775	2.135
*Clostridiaceae bacterium DJF LS13*	86.713	0.141	0.003	0.005	0.010	0.018	0.030	0.073	0.183	0.259	0.333
*Alistipes putredinis*	86.364	2.046	0.004	0.006	0.014	0.103	0.395	1.389	3.218	4.151	5.512
*Gemmiger formicilis (T)*	86.364	1.196	0.004	0.012	0.095	0.201	0.357	0.747	1.447	2.204	2.996
*Bacteroides* sp.	86.364	0.224	0.006	0.008	0.012	0.018	0.032	0.094	0.243	0.352	0.467
*Clostridium* sp. *enrichment culture clone 7-25*	86.014	0.419	0.007	0.015	0.029	0.041	0.076	0.238	0.552	0.794	0.997
*butyrate-producing bacterium A2-207*	85.664	0.137	0.005	0.006	0.010	0.015	0.023	0.057	0.166	0.265	0.358

**Figure 1:**
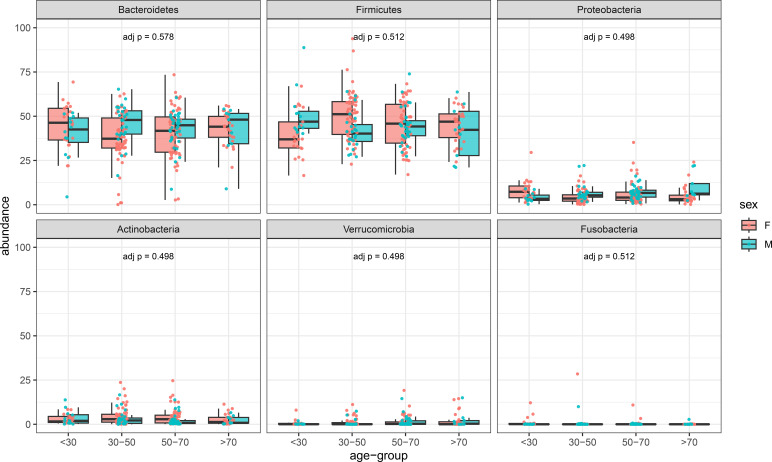
Prevalence of different phyla in females and males arranged for different ranges of age (horizontal axis). In the vertical axis, the prevalence of different taxa in percentage.

**Figure 2:**
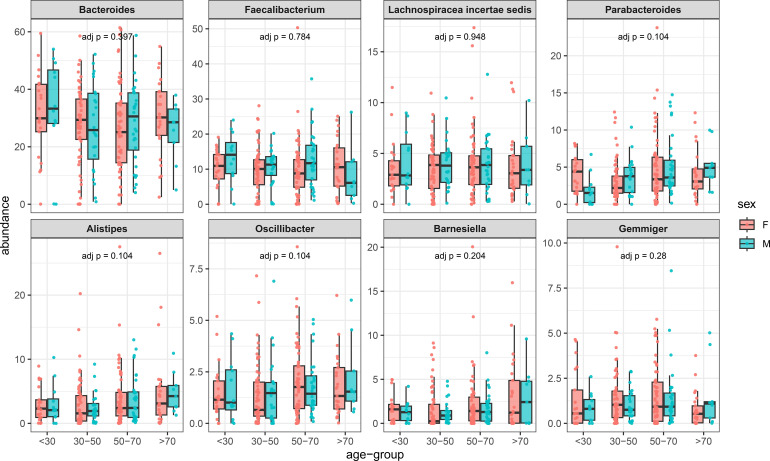
Prevalence of different genera in females and males arranged for different ranges of age (horizontal axis). In the vertical axis, the prevalence of different taxa in percentage.

**Figure 3:**
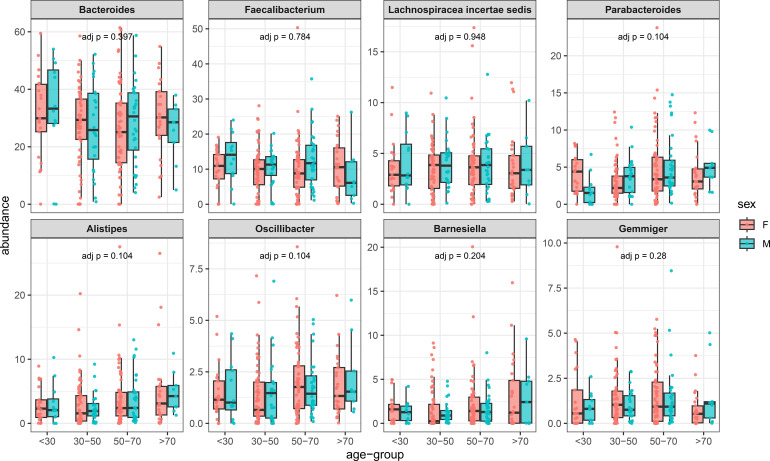
Prevalence of different species in females and males arranged for different ranges of age (horizontal axis). In the vertical axis, the prevalence of different taxa in percentage.

To further support the idea that these reference values can be considered useful, the mean values of representative phyla were calculated in different age intervals for males and females (**Figure 1**). According to the Kruskal-Wallis statistics, no differences were observed for these phyla. The *post hoc* Dunn test showed that for Proteobacteria, only a single comparison (F <30 vs F 30-50) was different (p=0.029). Similar results were also obtained when Genera and Species were analyzed (**Figures 2**, **3**).

Further quality controls were performed to assess the reliability of the experimental setup. Firstly, the reproducibility of the assay was evaluated through repeated analyses of the same stool sample, performed once per month throughout the two-year study period. The relative abundances of the major bacterial taxa at the phylum, class, and order levels were monitored. No parameter ever exceeded two standard deviations from the mean. Moreover, no parameter showed seven consecutive results above or below the mean, nor seven consecutive increasing or decreasing trends. Considering the intrinsic heterogeneity of fecal material, the complexity of NGS-based molecular assays, and their recent introduction into clinical diagnostics, these results confirm that the analytical system was stable and well controlled in terms of reproducibility.

**Figure 4:**
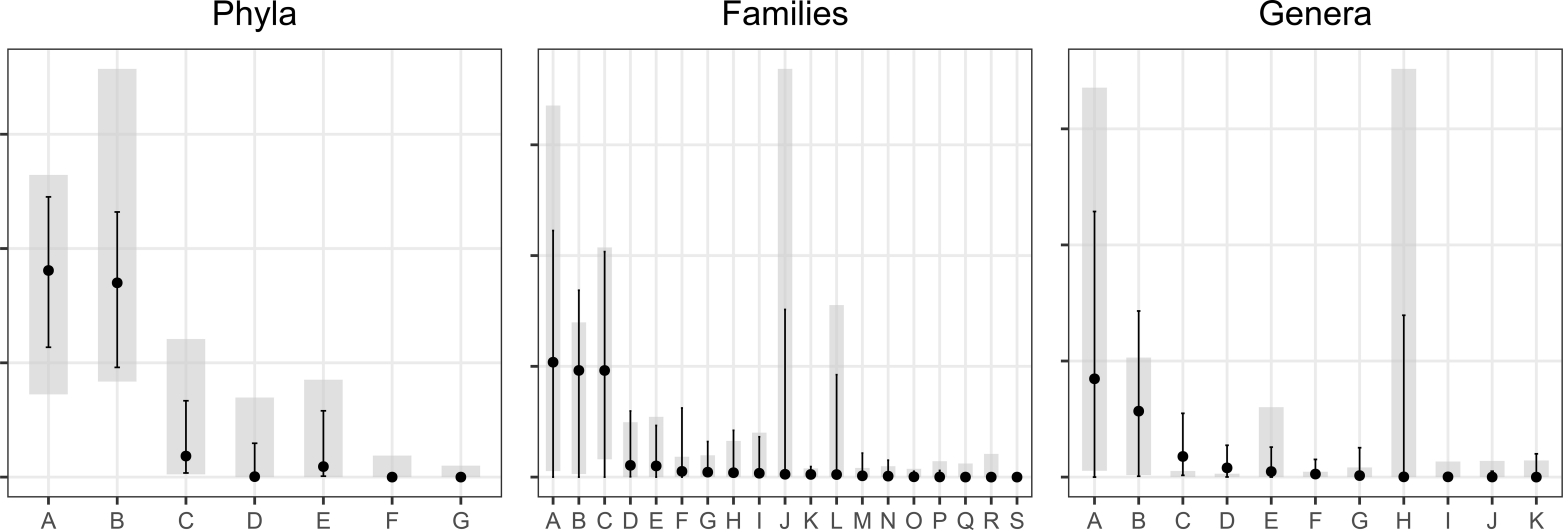
Comparison of relative abundances of different phyla, families and genera. Black dots indicate the median values from our dataset with vertical lines representing the 5th and 95th percentiles. Light gray rectangles show the reference distribution (5th–95th percentiles) reported in the Sisti et al. article; the short horizontal line within each rectangle corresponds to the reference median. Phyla: A, Firmicutes; B, Bacteroidetes; C, Proteobacteria; D, Cyanobacteria; E,Verrucomicrobia; F: Actinobacteria; G: Fusobacteria. Families: A, Bacteroidaceae; B, Ruminococcaceae; C, Lachnospiraceae; D, Rickenellaceae; E, Sutterrelaceae; F, Bifidobacteriaceae; G, Erysipelotrichaceae; H, Veilloneaceae; I, Addaminococcaceae; J- Prevotellaceae; K, Desulfovibrionaceae; L, Enterobacteriaceae; M- Streptococcaceae; N, Peptostreptococcaceae. Genera: A, Bacteroides; B, Faecalibacterium; C, Lachnospiraceae; D, OScillobacter; E, Roseburia; F, Corpobacter; G, Streptococcus; H, Prevotella; I, Holdemandia; J, Haemophilus; K, Addaminococcus. See the text for further explanations.

Then, the results of this study were compared with similar results obtained by another group in a similar population (Sisti et al., 2022). **Figure 4** shows the distribution of the 0.05 and 0.95 percentiles of the above-mentioned article (in light gray) and the distribution of the same percentiles in our study (in gray). The lines indicate the median values of the two studies. Virtually all results from our sample cohort were comprised in the range of values of the above-cited article. Only the family *Prevotellaceae* and the genus *Prevotella* were higher in the Sisti et al. group (2022) than in our study, although no clear explanation for this discrepancy can be proposed at this stage.

**Figure 5:**
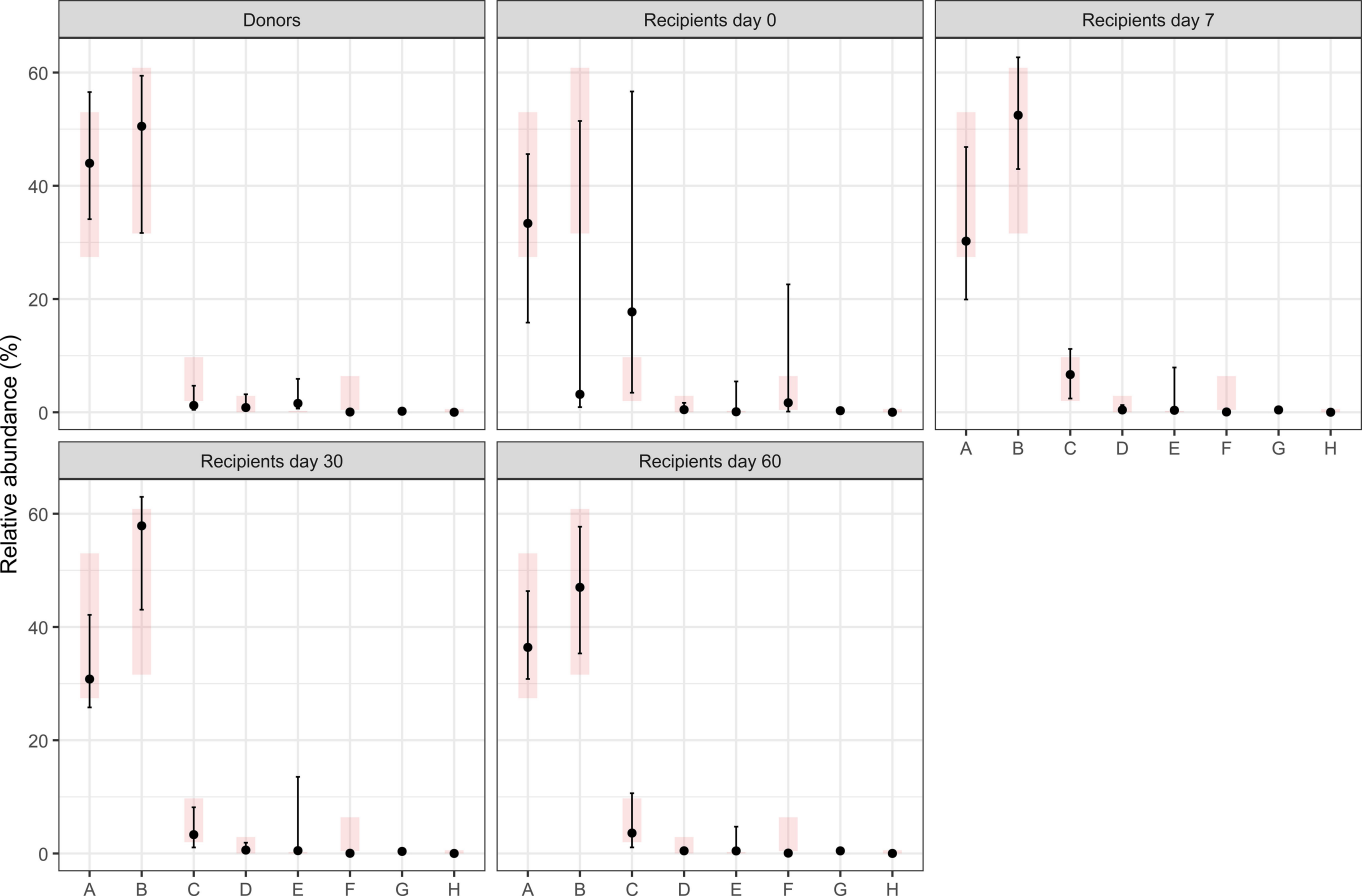
Comparison of the relative abundances of selected bacterial phyla in fecal transplant donors and recipients at different time points after FMT. Black dots indicate the median values from our dataset with vertical lines representing the 15th and 85th percentiles. Light gray rectangles show the reference distribution (15th–85th percentiles) reported in this article; the short horizontal line within each rectangle corresponds to the reference median. See the text for further explanations. A, Bacteroidetes; B, Firmicutes; C, Proteobacteria; D, Verrucomicrobia; E, Fusobacteria; G, Planctomycetes; H, Lentisphaerae.

**Figure 6:**
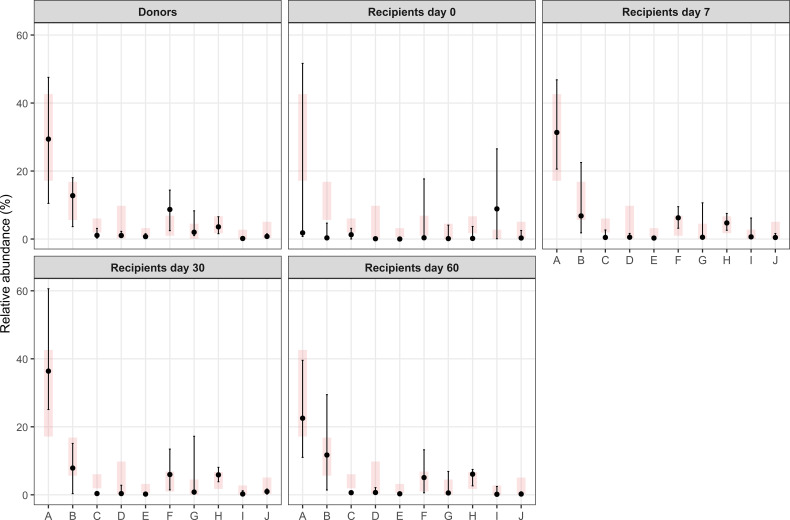
Comparison of the relative abundances of selected bacterial genera in fecal transplant donors and recipients at different time points after FMT. Black dots indicate the median values from our dataset with vertical lines representing the 15th and 85th percentiles. Light gray rectangles show the reference distribution (15th–85th percentiles) reported in this article; the short horizontal line within each rectangle corresponds to the reference median. See the text for further explanations. A, Bacteriodes; B, Faecalibacterium; C, Lachnospitaceae incertae sedis; D, Blautia; E, Oscillibacter; F, Alistipes; G, Akkermansia; H, Parabacteroides; I, Escherichia Shigella; J, Ruminococcus.

**Figure 7:**
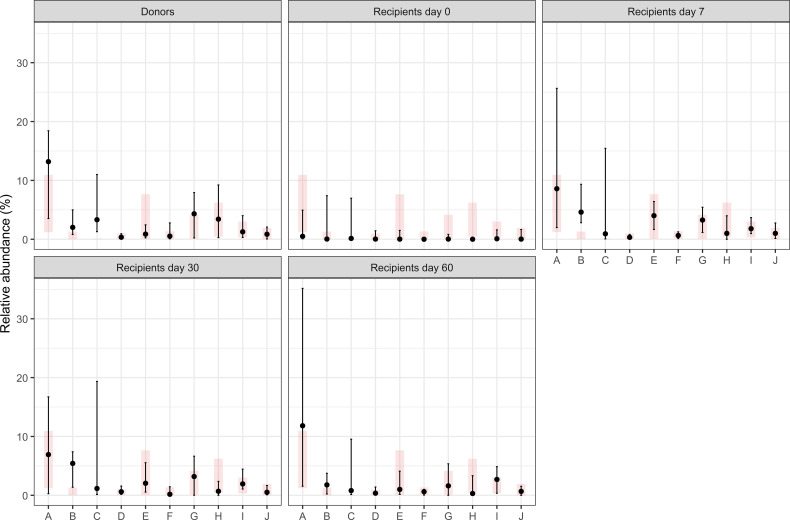
Comparison of the relative abundances of selected bacterial species in fecal transplant donors and recipients at different time points after FMT. Black dots indicate the median values from our dataset with vertical lines representing the 15th and 85th percentiles. Light gray rectangles show the reference distribution (15th–85th percentiles) reported in this article; the short horizontal line within each rectangle corresponds to the reference median. See the text for further explanations. A, Faecalibacterium prausnitzii; B, Bacterium mpn-isolate group 5; C, Akkermansia muciniphila; D, Bacteroides thetaiotaomicron; E, Bacteriodes vulfatus; F, Bacteroides uniformis; G, Alistipes putredinis; H, Bacteroides sp WA1; I, Parabacteroides distasonis; J, Bacteroides caccae.

Finally, in support of the concept that reference values, calculated in this report, have clinical significance, the results of Phyla analyzed in the context of a fecal transplant were used. **Figure 5** shows the distribution of the reference values (in pink) and the mean (and SD) of the results under the different conditions. It is evident that only samples obtained at time 0 from the transplant have a great difference with the reference values, while already after 7 days, results are much more comprised in the reference intervals. Similar results were also observed when Genera and species were analyzed (**Figures 6**, **7**).

## 4 Discussion

The analysis of the gut microbiota is progressively transitioning from research institutions to clinical practice. Assessing dysbiosis through an accurate characterization of bacterial communities across taxonomic levels enables gastroenterologists, dieticians, and nutritionists to obtain a detailed picture of the gut ecosystem. This, in turn, allows for personalized interventions ranging from dietary modifications to the administration of probiotics or pharmacological treatments (Shen et al., 2025).

However, several technical factors may influence the outcomes of next-generation sequencing (NGS) analyses, including sample collection, DNA extraction, indexing, and sequencing, all of which can vary depending on the reagents, instruments and applied protocols. Given the current lack of formal standardization, microbiota results remain only partially comparable across laboratories, limiting their direct applicability in clinical settings. The introduction of CE-certified reagents and platforms, compliant with EU regulatory standards, represents a key opportunity to harmonize procedures and establish reliable reference datasets. In this context, Statement 18 of the *International Consensus on Microbiome Testing* underscores that “appropriate comparison to a matched healthy control group should be included in microbiome testing to aid the interpretation of patient taxonomic and diversity profiles.” (Porcari et al., 2024). This recommendation highlights the urgent need for reference values across multiple taxonomic levels (such as Phyla, Classes, Orders, Families and selected Genera/Species) to support clinical interpretation. Literature data indicate that substantial heterogeneity exists across taxa, both at higher (phylum) and lower (species) levels, reflecting the strong influence of lifestyle, dietary habits, environment, and host-specific factors among individuals without gastrointestinal disease.

To address this, we analyzed the distribution of bacterial taxa in an unselected donor population, excluding only individuals reporting severe gastrointestinal symptoms, evident imbalances of the gut microbiota ecology or systemic metabolic diseases. This pragmatic approach mirrors strategies used in other areas of laboratory medicine where defining a “healthy” reference group can be challenging, and where representativeness and sample availability are critical. Our dataset showed a typical gut microbial structure dominated by Firmicutes and Bacteroidetes, with individual variability mainly driven by the balance between *Bacteroides*, *Prevotella* and *Faecalibacterium* lineages. The high dispersion in certain taxa such as *Akkermansia*, *Tenericutes* and *Proteobacteria* suggests the influence of host-specific or environmental factors (e.g., diet, health status, antibiotic exposure). The overall taxonomic composition aligns with a metabolically versatile microbiota capable of both saccharolytic and proteolytic functions, characteristic of human intestinal ecosystems.

A recurrent limitation of several microbiome studies is the reporting of only means or medians, without describing percentile-based intervals. Moreover, numerous studies rely on case-control designs comparing patients with specific diseases to small, unmatched control groups. While this design is appropriate for clinical research, it is less suited to routine diagnostics, where reference data should be representative across age and sex distributions. In a clinical context, the reference population effectively defines the measure of β-diversity, an essential parameter for identifying “abnormal” or “unexpected” microbiota profiles requiring further medical attention. As in other disciplines such as hematology, clinical chemistry and endocrinology, laboratory reports must include both reference intervals and the methods used to obtain them. Deviations from expected values, typically marked by symbols such as asterisks (*) or “H”/”L” labels, are key for clinical interpretation. Accordingly, to facilitate the translation of gut microbiota analysis into clinical practice, the establishment of reference values is mandatory. In this study, we utilized anonymized samples from our laboratory database to derive expected ranges for the principal bacterial taxa.

The advantages of this approach rely on the creation of reference values, useful to enable straightforward evaluation of β-diversity and contextual interpretation of individual patient profiles. In cases of microbiota imbalance, clinicians can make evidence-based decisions regarding lifestyle interventions, dietary changes, probiotic or prebiotic supplementation, pharmacotherapy, or even fecal microbiota transplantation.

Nevertheless, some limitations must be acknowledged. The present dataset, comprising 250 donors, does not provide sufficient statistical power to accurately estimate the frequencies of rare taxa (below 0.1% relative abundance). In this situation, the interpretation of the results should be clearly based on clinical evidence more than on the specific position out of reference ranges. Indeed, a larger dataset (exceeding 15, 000 samples) would be necessary to achieve stable estimates for the rarest species. As clearly shown in the first column of the percentile distribution of different taxa, many low-abundance taxa were detected in fewer than 20% samples, highlighting inherent variability and suggesting that approximately one-fifth of gut microbial communities may harbor highly individual, low-frequency species. In conclusion, this work provides reference values for gut microbiota taxa obtained using a CE-certified *in vitro* diagnostic (IVD) method. While other analytical platforms will require their own specific reference ranges, it is expected that results derived from standardized workflows will converge.

## Data Availability

The original contributions presented in the study are publicly available. This data can be found here: https://www.ncbi.nlm.nih.gov/sra/PRJNA1452189.
